# Effects of Shock and Vibration on Product Quality during Last-Mile Transportation of Ebola Vaccine under Refrigerated Conditions^1^

**DOI:** 10.3201/eid3004.231060

**Published:** 2024-04

**Authors:** Linda Bus-Jacobs, Rute Lau, Marjolein Soethoudt, Lisa Gebbia, Edwin Janssens, Tjeerd Hermans

**Affiliations:** Janssen Vaccines and Prevention B.V., Leiden, the Netherlands (L. Bus-Jacobs, R. Lau, M. Soethoudt, E. Janssens, T. Hermans);; Janssen Research and Development LLC, Spring House, Pennsylvania, USA (L. Gebbia)

**Keywords:** Ebola virus, Ebola virus disease, Ebola vaccine, vibration, vaccine potency, viruses, vaccines

## Abstract

Analyzing vaccine stability under different storage and transportation conditions is critical to ensure that effectiveness and safety are not affected by distribution. In a simulation of the last mile in the supply chain, we found that shock and vibration had no effect on Ad26.ZEBOV/MVA-BN-Filo Ebola vaccine regimen quality under refrigerated conditions.

Ebola vaccine development has been accelerated in response to large outbreaks in West and Central Africa; those outbreaks have caused >32,000 cases and >13,500 deaths (*1*). One vaccine emerging from this effort is the heterologous Ad26.ZEBOV/MVA-BN-Filo regimen (Johnson & Johnson, https://www.jnj.com). Ad26.ZEBOV, an adenovirus serotype 26–vectored monovalent, recombinant, replication-incompetent vaccine, encodes the full-length Ebola virus Mayinga glycoprotein. MVA-BN-Filo, a recombinant, nonreplicating, modified vaccinia Ankara–vectored multivalent vaccine, encodes the glycoprotein of Ebola virus (Mayinga), Sudan virus (Gulu), and Marburg virus (Musoke) and the nucleoprotein of Taï Forest virus. Ad26.ZEBOV/MVA-BN-Filo received marketing authorization under exceptional circumstances for prophylactic use in persons >1 year of age in the European Union (*2*,*3*) and is on the World Health Organization’s list of prequalified vaccines (*4*). This vaccine regimen has been shown to be safe and immunogenic in children and adults (*5*–*9*).

Recommended long-term storage and shipping conditions are –85°C to –55°C for the Ad26.ZEBOV component (*2*) and 25°C to –15°C for the MVA-BN-Filo component (*3*). However, infrastructure challenges may affect implementation of recommended distribution conditions, particularly in remote regions. Refrigerated (2°–8°C) liquid transport may more easily support extended vaccine distribution to rural locations than frozen conditions. Ad26.ZEBOV/MVA-BN-Filo can maintain stability at 2°–8°C (*10*). An important consideration in transporting liquid vaccines to rural areas is agitation, which can contribute to vaccine degradation and loss of potency (*11*). For our study, we subjected Ad26.ZEBOV/MVA-BN-Filo to simulated rough-road transport at 2°–8°C, conditions that are representative of the final leg or last mile in the supply chain in rural areas, to assess the effect of shock and vibration on vaccine quality.

## The Study

Packaging configurations (material used and shipping and storing conditions) were representative of supplies available in Africa during large vaccination campaigns (Figure; Appendix Table 1). We conducted simulated distribution testing in 2 sequential steps at 2°–8°C to approximate last-mile transport in rural areas that included a shock test followed by a vibration test. The test simulated unpaved roads typical of rural areas, representing long-distance (100-km) travel that could occur between the distribution center and vaccination site, by using a rough-road transport simulation profile.

**Figure:**
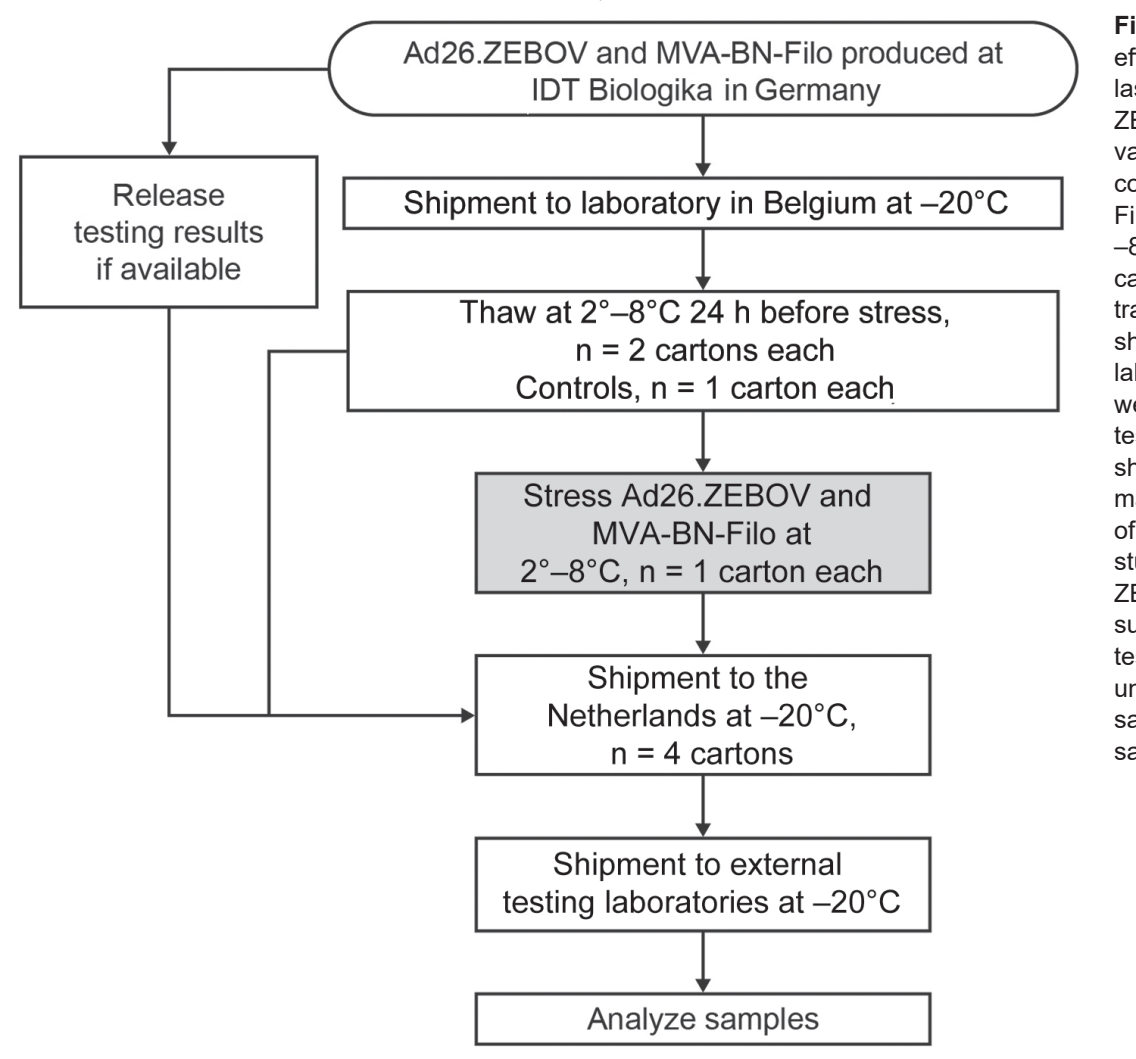
Study design to assess effects of shock and vibration on last-mile transportation of Ad26.ZEBOV/MVA-BN-Filo regimen Ebola vaccine regimen under refrigerated conditions. Ad26.ZEBOV/MVA-BN-Filo were produced and stored at –85°C to –55°C. Four paperboard cartons, each with 2 thermoformed trays containing 10 vials, were shipped to the simulation test laboratory at –20°C. The vials were thawed at 2*°*–8°C 24 h before testing and packed into insulated shipping containers designed to maintain an internal temperature of 2*°*–8°C for the duration of the study. Half of the vials (20 Ad26.ZEBOV and 20 MVA-BN-Filo) were subjected to simulated distribution testing, and half remained unstressed as controls. Control samples are non–distribution-tested samples exposed to freeze-thaw.

We dropped vials 9 times in various orientations from heights of 30.5–91.4 cm, depending on the weight of the shipping container (Appendix Table 2, Figure). We determined the vibration sequence by using the International Safe Transit Association’s web-based software application 4AB (https://ista.org/test_procedures.php#enhanced-simulation-section), selecting the truck profile to approximate truck transport in rural areas. During the 36-minute vibration test, vibration intensity increased and the vibration profile shape remained constant. Vials were oriented upside down to simulate a worst-case scenario.

After simulated distribution testing, we froze all 80 vials at –20°C and shipped them back to analytical laboratories, where they were stored at –85°C to –55°C before analysis. We applied assays for indicating critical quality attributes, including appearance, potency, quantity, aggregation, protein impurities, and presence of subvisible particles (Appendix Table 3). We conducted potency testing as described previously (*10*). We selected test methods and release specifications on the basis of regulatory guidance provided in the International Conference on Harmonisation Q6B and European Pharmacopoeia (*12*,*13*).

We compared assay results from the drug product vials with the control (unstressed) vials from the same batch of drug product and the release specifications, noting any deviations that occurred during the study (Appendix). The attributes evaluated did not show substantial differences between stressed and control samples of Ad26.ZEBOV (Appendix Table 4). After the simulation, the appearance of the Ad26.ZEBOV drug product was clear, without coloration or visible particulate matter. Potency, quantity of Ad26.ZEBOV viral particles, and polydispersity were within release specifications. We observed no difference in the average hydrodynamic radius of stressed (54.2 nm) and control (55.2 nm) samples. Those values exceeded the current release specification (<53 nm), which is attributable to the method used for this study having an ≈5 nm bias versus the release method. The percentage of main hexon identified on reverse phase ultra-high performance liquid chromatograms was numerically lower in both stressed (65.06%) and control (64.42%) samples versus the reference batch (75.1%). The percentage of free hexon was similar in stressed (4.3%) samples and the reference batch (4.0%).

We observed no substantial effect on attributes of the MVA-BN-Filo drug product between stressed and control samples after the simulation (Appendix Table 4). The appearance of material in both groups was light yellow and milky, with no visible extraneous particles; product-related particles were present in the stressed vials. Potencies of stressed and control samples were within commercial release specifications. We confirmed the transgene expression of the Zaire Ebola virus (Mayinga), Sudan virus (Gulu), and Marburg virus (Musoke) glycoproteins and the Taï Forest virus nucleoprotein encoded by MVA-BN-Filo in stressed and control samples. Genomic vaccinia DNA contents were identical between the 2 groups. Average particle sizes measured by nanoparticle tracking analysis were numerically higher in stressed (195 nm) and control (191 nm) samples versus the reference batch (157 nm). Average virus particle sizes measured by fluorescence nanoparticle tracking analysis were numerically higher in the stressed samples (453 nm) versus both the control samples (399 nm) and the reference batch (394 nm). Total subvisible particle concentrations measured by microflow imaging were numerically higher in stressed (7.82 × 10^6^ particles/mL) versus control (4.73 × 10^6^ particles/mL) samples, whereas mean subvisible particle sizes were numerically lower in stressed (2.49 μm) versus control (3.26 μm) samples. We identified no substantial differences between stressed and control samples from the particle size distribution graphs of the 3 methods.

## Conclusions

The stability of Ebola virus vaccine drug products is critical to ensure that quality remains unaffected in conditions likely encountered during distribution. We have shown that a liquid formulation for Ad26.ZEBOV/MVA-BN-Filo is suitable for mass vaccination in resource-limited regions at risk for outbreaks (*10*). We assessed the impact of shock and vibration, simulating rough-road transport conditions at 2°‒8°C, on the quality of Ad26.ZEBOV/MVA-BN-Filo.

Product stability can be demonstrated through a quantitative measure of potency, which is linked to vaccine safety and efficacy (*14*). Potency can correlate with infectious titer and transgene expression and may be negatively affected by aggregate formation (*14*). Aggregates can affect immunogenicity and subsequently compromise vaccine efficacy (*14*). We have demonstrated that Ad26.ZEBOV and MVA-BN-Filo undergoing simulated distribution contained similarly sized aggregates and viral particles similar in number and potency to the control samples, suggesting that the immunogenicity of samples exposed to simulated rough-road transport would not differ from the control samples.

One limitation of this study is that the stress that may be encountered during in-country transport was simulated. However, the simulation used in this study provides more realistic exposure to shock and vibration than can be generated by laboratory orbital shakers. Data on stability after agitation of alternative candidate Ebola virus vaccine regimens are limited in this regard (*15*).

In summary, this study shows that simulated, refrigerated (2°–8°C), rough-road truck transport, representative of the last mile of the supply chain, had no substantial effect on the quality of Ad26.ZEBOV/MVA-BN-Filo compared with the control condition. These findings confirm that refrigerated transport over rough terrain is possible and meets requirements for the challenging rural areas in the cold chain.

Appendix Additional information about effects of shock and vibration on product quality during last-mile transportation of Ebola vaccine under refrigerated conditions.
