# Supplementary material for: Effects of Shock and Vibration on Product Quality during Last-Mile Transportation of Ebola Vaccine under Refrigerated Conditions
Source: Emerg Infect Dis. 2024 Apr;30(4):757–60. doi: 10.3201/eid3004.231060 (PMC10977826; doi:10.3201/eid3004.231060)
Supplement: Appendix — Additional information about effects of shock and vibration on product quality during last-mile transportation of Ebola vaccine under refrigerated conditions. [file 23-1060-Techapp-s1.pdf]

Article DOI: https://doi.org/10.3201/eid3004.231060

Effects of Shock and Vibration on Product Quality during Last-Mile Transportation of Ebola Vaccine under Refrigerated Conditions

Appendix

Supplemental Results

Protocol deviations

The first deviation involved the test laboratory inadvertently subjecting all 80 vials of Ad26.ZEBOV and MVA-BN-Filo to the distribution test sequence without reserving 20 vials of each drug product as controls. Twenty additional vials of each product from the same manufacturing lots were shipped to the analytical laboratory at –20°C, where they were thawed at 2°–8°C for 24 hours and returned to –20°C to mimic what would have happened at the simulation test laboratory. These vials were then sent for analysis. This deviation was considered to have no impact on the results of the study.

The second deviation occurred due to inadequate sample volume for reverse phase high performance liquid chromatography (RP-HPLC). A volume of ≥1.5 mL is required, but this amount was not available for the control samples. Due to the first deviation, there were 20 extra vials of the stressed sample from which adequate volume could be derived to perform free hexon quantification via RP-HPLC.

The third deviation involved invalid runs of the real-time polymerase chain reaction-based potency assay (QPA), which resulted in the need for retesting. However, there were insufficient backup samples available to rerun the assay. Given that it is known that 1 freeze-thaw cycle does not impact the product, it was determined that leftover samples could be used for retesting of the invalid runs.

Appendix Table 1. Materials used for packing and shipment of Ad26.ZEBOV and MVA-BN-Filo vaccine drug products. The target fill volume was 0.69 mL in a 2-mL vial

Material	Vial configuration
Ad26.ZEBOV	
Vial	Schott (type I glass)
Stopper	13 mm West 4432/50 (rubber)
Seal	13 mm West (aluminum flip off)
MVA-BN-Filo	
Vial	Thüringer Pharmaglas (type I glass)
Stopper	13 mm Datwyler FM457 (rubber)
Seal	13 mm West (aluminum flip off)

Appendix Table 2. Shock (drop) test sequence and parameters based on the International Safe Transit Association's software 4AB

Drop (orientation)*	Drop height, cm (31.8–68.0 kg)
1 (edge 3–4)	30.5
2 (edge 3–6)	30.5
3 (edge 4–6)	30.5
4 (corner 3–4–6)	30.5
5 (corner 2–3–5)	30.5
6 (edge 2–3)	30.5
7 (edge 1–2)	30.5
8 (face 3)	61.0
9 (face 3)	30.5

*Orientation of the packing materials is described in Appendix Figure.

Appendix Table 3. Analytical panel for analyzing Ad26.ZEBOV and MVA-BN-Filo drug products subjected to simulated distribution testing*

Attribute/assay	Test volume	Stressed vials (n)	Control vials (n)	Assay format (run × replicates)
Ad26.ZEBOV				
Appearance				
Degree of coloration<br>Clarity<br>Visible particles	6000 µL	9	9	–
Potency				
Infectious units by QPA	<500 µL	4†	4†	3 × 3
Quantity				
Virus particles by vp-qPCR	10 µL	4†	4†	3 × 3
Impurities/aggregates				
Average hydrodynamic aggregate radius by DLS<br>Polydispersity by DLS	>250 µL	1	1	–
Protein profile/impurities				
Viral protein degradation products by RP-UPLC	<500 µL	1	1	–
Impurities				
Free hexon by RP-HPLC	≥1500 µL	1	1	–
MVA-BN-Filo				
Appearance				
Degree of coloration<br>Clarity<br>Visible particles	1 vial	2†	2†	–
Potency				
Infectious units by FACS<br>Quantitative transgene expression (GP-Z-EBOV, GP-S-EBOV, NP-IC-EBOV, GP-MARV-MU)	500 µL	4†	4†	3 × 1
Quantity				
Quantification of genomic vaccinia DNA	1 vial	2†	2†	–
Aggregation				
NTA	1 vial	2†	2†	–
Virus particle aggregation				
Fluorescence NTA	1 vial	2†	2†	–
Subvisible particle aggregation				
MFI	1 vial	2†	2†	–

*DLS, dynamic light scatter; FACS, fluorescence-activated cell sorting; MFI, microflow imaging; NTA, nanoparticle tracking analysis; QPA, real-time polymerase chain reaction-based potency assay; RP-HPLC, reverse phase high-performance liquid chromatography; RP-UPLC, reverse phase ultra-performance liquid chromatography; VP-qPCR, virus particle real-time polymerase chain reaction.
†Includes 1 vial available for backup.

Appendix Table 4. Analytical test results for Ad26.ZEBOV and MVA-BN-Filo samples subjected to simulated distribution testing*

			Study results		
Attribute	Limit	Reference batch	Stressed	Control	Conclusion†
Ad26.ZEBOV					
Appearance					
Degree of coloration	<Reference solution B9, BY7, Y7, and GY7‡	<Reference solution B9, BY7, Y7, and GY7‡	<Reference solution B9, BY7, Y7, and GY7‡	<Reference solution B9, BY7, Y7, and GY7‡	Pass
Clarity	<Reference suspension III‡	<Reference suspension III‡	<Reference suspension III‡	<Reference suspension III‡	Pass
Visible particles	Essentially free of visible particulate matter	Essentially free of visible particulate matter	Essentially free of visible particulate matter	Essentially free of visible particulate matter	Pass
Potency					
Infectious units, $\log_{10}$ Inf.U/mL	≥9.30	9.55	9.48 (0.11)	9.49 (0.14)	Pass
Quantity					
Virus particles, vp × 10^{11}/mL	0.5–2.0	0.8	0.81 (0.15)	0.74 (0.14)	Pass
Impurities/aggregates					
Aggregate radius, nm	≤53	53	54.2	55.2	No substantial difference
Polydispersity, %	≤25	7.7	6.4	6.9	No substantial difference
Protein profile/impurities					
Main hexon, %	–	75.1	65.06	64.42	No substantial difference
Unidentified peaks, %	–	4.2	3.06	2.99	No substantial difference
Impurities					
Free hexon, %	–	4	4.3	ND§	No substantial impact
MVA-BN-Filo					
Appearance					
Degree of coloration	Light yellow	Light yellow	Light yellow	Light yellow	Pass
Clarity	Milky	Milky	Milky (100–200 NTU)	Milky (100–200 NTU)	Pass
Visible particles	Homogenous suspension	Homogenous suspension	No visible extraneous particles but product-related particles present	No visible extraneous particles	Pass
Potency					
Infectious units, Inf.U × 10^{8}/mL	2.45–5.34	2.49	3.36 (0.26)	3.05 (0.47)	Pass
Transgene expression, TxgU/Inf.U	Expression confirmed				Pass
ZEBOV		0.58	0.59 (0.06)	0.63 (0.12)	
MARV		0.47	0.55 (0.04)	0.58 (0.04)	
NP		1.53	1.43 (0.07)	1.44 (0.06)	
SEBOV		0.54	0.48 (0.05)	0.49 (0.08)	
Coexpression, %		ND	45.5 (3.5)	45.5 (3.5)	Pass
Quantity					
Genomic vaccinia DNA, molecules × 10^{9}/mL	–	ND	1.70	1.70	No substantial difference
Aggregation					
Total particles × 10^{11}/mL	–	ND	1.48	1.41	No substantial difference
Aggregate size, nm	–	157 (6)	195	191	No substantial difference
Virus particle aggregation					
Total particles × 10^{9}/mL	–	ND	5.66	5.52	No substantial difference
Aggregate size, nm	–	394 (42)	453	399	No substantial difference
Subvisible particle aggregation					

Attribute	Limit	Reference batch	Study results		
			Stressed	Control	Conclusion†
Total particles × 10^6/mL	–	ND	7.82	4.73	No substantial difference
Aggregate size, µm	–	ND	2.49	3.26	No substantial difference

*Data are presented as mean or mean (SD). B, brown; BY, brownish-yellow; GY, greenish-yellow; Inf.U, infectious units; ND, not determined; NTU, nephelometric turbidity units; TxgU, transgene units; Y, yellow.

†Conclusion is based on the result of a stressed sample analyzed by itself (no substantial t impact), in comparison with control (no substantial difference), or in comparison with commercial release specifications in cases of critical quality attributes (pass). A pass automatically signifies no difference between stressed and control samples.

‡Color reference solutions and reference suspensions are described in monograph 2.2.1 “Clarity and degree of opalescence of liquids” and 2.2.2 “Degree of coloration of liquids” of the European Pharmacopeia (https://www.edqm.eu/en/d/99080).

§A minimum volume of 1.5 mL was not available for control samples.

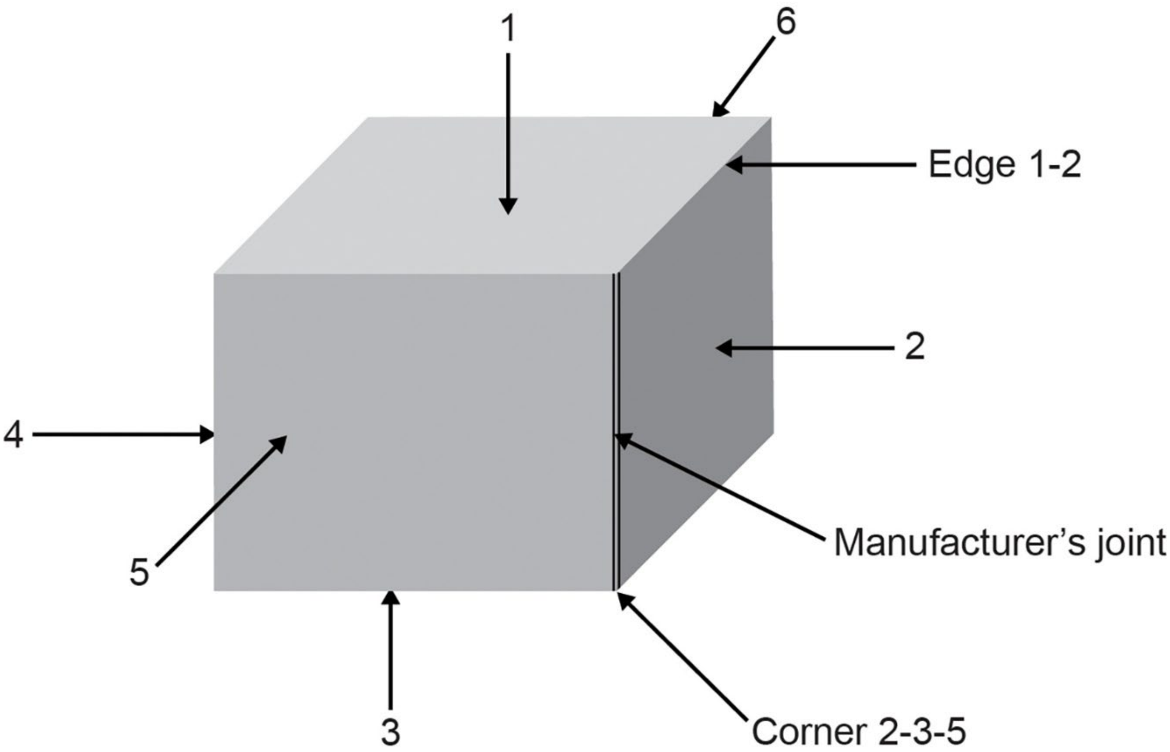

Appendix Figure. Orientation of packing materials during simulated distribution testing.
